# Influence of online collaborative learning on social network and academic performance of medical students: lessons learned from the COVID-19 pandemic

**DOI:** 10.3389/fmed.2023.1242638

**Published:** 2023-08-09

**Authors:** Yan Zhou, Xiaoming Xu, Johanna Schönrock-Adema, Jasperina Brouwer, Nicolaas A. Bos, Agnes D. Diemers

**Affiliations:** ^1^Key Laboratory of Intelligent Education Technology and Application of Zhejiang Province, Zhejiang Normal University, Jinhua, Zhejiang, China; ^2^Wenckebach Institute for Education and Training, University of Groningen, University Medical Center Groningen, Groningen, Netherlands; ^3^International Institutes of Medicine, Zhejiang University, Yiwu, Zhejiang, China; ^4^The Fourth Affiliated Hospital, Zhejiang University School of Medicine, Yiwu, Zhejiang, China; ^5^Prins Claus Conservatorium, Hanze University of Applied Sciences, Groningen, Netherlands; ^6^Educational Sciences, Faculty Behavioural and Social Sciences, University of Groningen, Groningen, Netherlands

**Keywords:** social networks, online learning, medical students, COVID-19 pandemic, academic performance

## Abstract

**Introduction:**

The social distancing restrictions due to the COVID-19 pandemic have changed students’ learning environment and limited their social interactions. Therefore, the objective of this study was to investigate the influence of the social distancing restrictions on students’ social networks, wellbeing, and academic performance.

**Methods:**

We performed a questionnaire study in which 102 students participated before and 167 students during the pandemic. They completed an online questionnaire about how they formed their five peer social networks (study-related support, collaboration, friendship, share information, and learn-from) out-of-class. We performed social network analysis to compare the sizes, structures, and compositions of students’ five social networks before and during the pandemic, between first- and second-year students, and between international and domestic students. Additionally, we performed Kruskal–Wallis *H* test to compare students’ academic performance before and during the pandemic. We performed thematic analysis to answers for two open-end questions in the online questionnaire to explore what difficulties students encountered during the COVID-19 pandemic and what support they needed.

**Results:**

The results showed that the size of students’ social networks during the pandemic was significantly smaller than before the pandemic. Besides, the formation of social networks differed between first- and second-year students, and between domestic and international students. However, academic performance did not decline during the COVID-19 pandemic. Furthermore, we identified three key areas in which students experienced difficulties and needed support by thematic analysis: social connections and interactions, learning and studying, and physical and mental wellbeing.

**Conclusion:**

When institutions implement learning with social distancing, such as online learning, they need to consider changes in students’ social networks and provide appropriate support.

## Introduction

The COVID-19 pandemic not only had a significant impact on clinical care delivery, but also on the structure and delivery of medical education (1–4). Social distancing to control the COVID-19 pandemic limited social interactions, increased the risk of social isolation among students and, therefore, may have negatively influenced their social network formation and mental health (5, 6). It is well-known that social interactions strongly influence students’ learning engagement and academic performance (6–8). As with MOOCs (Massive Open Online Courses), where students in the same course can only contact fellow students by the Internet instead of face-to-face, the larger transactional distance may increase the communication cost and lead to low engagement and high demands on students’ motivation (9–11). Especially first-year university students who do not yet have social networks and start their studies during a pandemic requiring social distancing may encounter difficulties in establishing social networks and need additional support to help them cope with their new learning environment (12). To enhance educational institutions’ readiness for future situations in which students must maintain social distancing, it is important to know whether and how social distancing relates to medical students’ social network formation and academic performance.

Good relationships are considered one of the key elements of human environments in general and, more specifically, of qualitative good learning environments, in addition to goal direction and proper organization/regulation (13–15). Besides, medical students need to train their competencies described in the CanMEDS framework to become good collaborators, scholars, and medical experts (16). In other words, medical students need to be lifelong learners and good team workers who are able to apply medical knowledge and clinical skills, willing to share their knowledge and experience, able to seek help from others, and learn from others to improve themselves. Interaction between students may be a prerequisite for developing these professional competencies: building good relationships with peers can help students function more effectively and facilitate their competency development and achievement of academic goals they would not have achieved otherwise (17–21). However, at times when social distancing is required and opportunities for social interactions are limited, students have fewer opportunities to reach out and build social networks, which in turn may negatively affect their competency development and mental health (6, 7, 21–24). The fact that the same group activities were designed both on-site (before-pandemic) and online (during-pandemic) allowed us to investigate the impact of social distancing on the formation of several types of social networks relevant to the development of medical students’ competencies and knowledge. Therefore, we aimed to investigate whether the social distancing measures actually limited the formation of social networks and students’ learning outcomes.

According to current literature, five types of social networks appear to be most relevant to medical students for developing competencies defined within the CanMEDS framework: (1) study-related support (help-seeking relationship within the study institution) (25–28); (2) collaboration (academic collaboration for assignments or course-related tasks with peers from the same study institution) (29, 30); (3) friendship (friends from the same study institution) (31–33); (4) information-sharing (study-related information sharing with peers from the same study institution) (9, 31); and (5) learn-from (peer role model from the same study institution) (27, 32). Existing studies on medical education during COVID-19 have focused on students’ perceptions of the pandemic (1, 34), technical resources, and differential access to online education (34–36), however, they rarely addressed the formation of social networks. One of the few exemptions is the study by Elmer et al. (6), showing that interpersonal interactions and co-study networks became sparser during the pandemic and that more students were isolated in social networks (6). However, when students participate in online learning, social interaction positively impacts their self-directiveness via emotional and behavioral engagement aspects (37). It is necessary to understand how students’ social networks form when they have social distancing and to what extent their attributes relate to it. To our knowledge, research so far has rarely focused on how COVID-19 influenced the formation of different types of social networks. This study contributes to filling this gap by exploring the impact of social distancing with a focus on five types of social networks that may affect student learning and wellbeing in different ways.

Meanwhile, we are aware of possible differences between domestic and international students. On top of the challenges all students face in their academic life, a transition to online education and social distancing can have a negative impact on their social networks (36–38). However, the impact may even be larger for international students who come to a foreign country where they experience language barriers and cultural differences (39, 40). Considering the challenges international students already face in building social networks with domestic students (41, 42), social distancing may even make it harder for them to build social relationships.

In a prior study, we performed just before the pandemic struck, we investigated social network formation and academic performance. Repeating this study during the pandemic would offer us a unique opportunity to examine whether and how social network formation and academic performance developed differently when students meet social distancing. In this study, we investigated to what extent social network formation during the pandemic differed between first- and second-year students, and between domestic and international students. Furthermore, using open-ended questions we investigated what difficulties students experienced when they studied and contacted others only online during the pandemic and what support they needed. In sum, our research questions were: (1) How did social distancing influence the social network formation and academic performance of first-year and second-year domestic and international medical students? (2) Which difficulties did students encounter during the COVID-19 pandemic and what kind of support did they need? We hoped that our research would shed light on the changes and challenges social distancing measures brought to medical students and provide recommendations for educational institutions to help their students thrive in a changing learning environment.

## Materials and methods

### Participants and data collection

This study was conducted in an undergraduate curriculum of a University Medical School in Netherlands. To explore the influence of social distancing on students’ social networks and academic performances, we compared social networks and academic performances of first- and second-year undergraduate students between the before-pandemic (in the academic year 2018-2019) and the during-pandemic cohorts (in the academic year 2020–2021). In 2019, 59 first-year and 43 second-year students participated, and in 2021, 102 first-year and 65 second-year students participated in this mixed methods social network study (Table 1). Notably, social distancing may be more problematic for the first-year students of the during-pandemic cohort since their first-year study took place almost entirely online. The second-year students, however, followed only part of their program online as their first-year study was on campus for half a year. After that, they followed the second half of the first academic year and the second year, 2020–2021, mostly online.

**TABLE 1:** Participant cohorts and demographics.

Students before the pandemic (N = 102)	First-year (BA1819)	Second-year
Total	59	43
Female (%)	50 (84.75)	31 (72.09)
Male (%)	9 (15.25)	12 (27.91)
Domestic (%)	35 (59.32)	35 (81.40)
International (%)	24 (40.68)	8 (18.60)
**Students during the pandemic (N = 167)**	**First-year (BA2021)**	**Second-year**
Total	102	65
Female (%)	79 (77.45)	55 (84.62)
Male (%)	23 (22.55)	10 (15.38)
Domestic (%)	81 (79.41)	47 (72.31)
International (%)	21 (20.59)	18 (27.69)

We collected three types of data. First, we used an online questionnaire to collect quantitative data about students’ social networks (see Supplementary Table 1). We asked students to fill in their IDs when filling out the questionnaire. In both 2019 and 2021, all participants received our online questionnaire via email. Secondly, using the ID information that participants provided in this online questionnaire, we collected data through the administration of the medical school on participants’ academic performance and on whether they were domestic students or international students. We used student IDs to match students’ nationalities, academic performance, and their social networks. Finally, we collected qualitative data using open-ended questions from the during-pandemic cohort about how students perceived the impact of COVID-19 on their interpersonal contacts and wellbeing.

### Instruments

#### Social networks

We focused our study on five types of social networks, namely, study-related support, collaboration, friendship, information-sharing, and “learned-from” networks. To collect data about these five types of informal networks, we used the questionnaire by Zhou et al. (43), which was based on previous social network research (25, 43, 44) (see Supplementary Table 1). An example of a study-related support social network question is: “In the last semester, when I didn’t understand the study material, I discussed this with following senior students/teachers outside of class.” We asked students to fill in the names of fellow students with whom they had had regular contact outside of class during the past semester to indicate who they connected with for each of the five types of social networks. Students needed to type in the names of peers (up to a maximum of 25) they contacted. Students’ interactions with each other outside of curricular activities represent the types of social networks they were involved in.

#### Academic performance

We used written test scores as a measure of students’ academic performance. These written test scores came from curriculum-dependent tests to assess students’ medical knowledge. Per semester, students took 4–5 written tests consisting of multiple-choice questions of which the scores were aggregated to an overall test score at the end of that semester. The cut-off values were corrected for the variation in test difficulty by applying the Cohen-Schotanus method using the 1% best-scoring students as reference for the maximum score (45). The written test scores ranged from 1 (very poor) to 10 (excellent), with a passing score of 5.5 or higher. This study presented students’ academic performance in terms of semester average scores at the time point of the survey. All written tests, both before- and during-pandemic cohorts, were administered offline.

#### Domestic versus international students

We investigated whether the restrictions affected domestic and international students differently. We defined international students in this study as the students who moved to the Netherlands for their bachelor’s degree programme in Medicine.

### Ethical approval

The before-pandemic social network study was approved by the Ethical Review Board of the Netherlands Association of Medical Education (NVMO), dossier number 2019.4.9. Ethical approval of during-pandemic survey was obtained from the Central Ethics Committee of the University Medical Center of Groningen (No. 20200869). Participation was voluntary and participants gave consent before answering the online questionnaire. Participants were also asked for informed consent to couple their academic results to their research ID.

### Data analysis

We used mixed methods to investigate the COVID-19 pandemic influences. Firstly, we investigated whether social distancing influences first- and second-year students’ social network sizes and structures differently. Individual social network size in this study represents how many contacts the student has (the number of nominations provided). We calculated and compared the number using ANOVA. Besides, we conducted descriptive Social Network Analysis (SNA) in terms of degree centrality and investigated the social network structure differences by calculating and comparing the indegree and outdegree centrality. The indegree centrality presents incoming connections (popularity) while the outdegree centrality presents outgoing connections (activity) (46). Higher centrality refers to individuals who have a more dominant position in their social networks, which means that some individuals are influencers, popular, and connected to many others.

Secondly, we investigated whether social distancing has different effects on domestic and international students in terms of social network size and composition by comparing their individual network sizes and the External-Internal (E-I) index between domestic and international students. The E-I index measures similarity by comparing the number of connections of group members with the same background characteristics with those of group members with different background characteristics (47). The index ranges from −1 (all connections are within domestic or international students) to + 1 (all connections are between domestic and international students) (48). We used the UCINET 6.690 software package (Analytic Technologies, Harvard, MA, USA, 2002) to perform the network analysis (49).

Thirdly, to compare medical students’ academic performance between before- and during-pandemic cohorts, we analyzed the written test scores of all first- and second-year students using the Kruskal–Wallis *H* test, since their written test scores were not normally distributed. The SPSS 26.0 program was used for academic performance comparison (50).

At last, to understand what difficulties students encountered during the COVID-19 pandemic and what support they needed, we used thematic analysis according to Braun and Clark to analyze students’ answers to open-ended questions (51). Firstly, two members of our research team (YZ and XX) read all students’ answers to familiarize themselves with the data. Then, YZ and XX generated initial themes and discussed the potential themes with other members of the research team (YZ, XX, AD, and NB), during which we established preliminary themes before coding. Consecutively, YZ and XX started coding the open-ended questions iteratively and independently by use of ATLAS.ti (52) Web (Version 3.15.0-2022-03-09). After coding 20 students’ answers, YZ and XX discussed their codes until they reached a consensus about the codes. To ensure the reliability of the code, they were discussed with another team member (AD). Then YZ and XX coded another 20 students’ answers and again discussed the codes until they reached a consensus within the team. They repeated this process until they established the final coding tree and coded all students’ answers accordingly. After coding students’ answers, YZ and XX determined if the codes suited the preliminary defined themes. To ensure consistency, AD once more reviewed the codes and the themes. YZ, XX, and AD finally discussed the findings which resulted in 3 main themes with their subthemes. These were discussed within the research team and supported by illustrative quotes.

## Results

### Social network size before and during the COVID-19 pandemic

#### Social network size comparison between first- and second-year medical students

The investigation into social network size showed that in general, first- and second-year medical students from the during-pandemic cohorts had significantly smaller individual network sizes than students from the before-pandemic cohorts [see Table 2; *p* < 0.05 for each of the networks, with medium or large effects (Supplementary Table 2)]. Closer inspection shows that before the pandemic, first-year students had larger networks than second-year students, except for the collaboration network, whereas during the pandemic, first- and second-year students tended to have social networks of similar sizes. Comparing the five types of social networks, the friendship network was the largest one before the pandemic, while during the pandemic, the largest network was the study-related support network.

**TABLE 2:** Average individual network size of students before and during COVID-19 considering students’ nationalities.

		Study-related support		Collaboration		Friendship		Information sharing		Learn-from	
		**Before**	**During**	**Before**	**During**	**Before**	**During**	**Before**	**During**	**Before**	**During**
Y1	Total, mean (SD)	6.19* (4.35)	3.92 (2.60)	3.93 (2.83)	2.70 (2.20)	6.81 (5.56)	3.18 (3.09)	6.25 (5.18)	3.35 (3.67)	3.76 (4.30)	2.06 (2.17)
	Domestic, mean (SD)	6.51 (3.71)	4.10 (2.80)	3.86 (2.70)	2.65 (2.29)	5.54 (2.98)	2.90 (3.05)	5.37 (4.07)	3.21 (3.65)	2.80 (2.54)	2.15 (2.26)
	International, mean (SD)	5.71 (5.11)	3.24 (1.38)	4.04 (3.02)	2.86 (1.75)	8.67 (7.57)	4.24 (2.94)	7.54 (6.25)	3.90 (3.61)	5.17 (5.73)	1.71 (1.80)
Y2	Total, mean (SD)	5.91 (4.50)	3.92 (3.05)	4.49 (3.14)	2.35 (2.02)	6.51 (5.19)	3.45 (2.71)	5.28 (4.67)	3.37 (3.07)	3.70 (3.74)	1.88 (2.40)
	Domestic, mean (SD)	6.17 (4.74)	4.11 (3.25)	4.54 (3.22)	2.32 (1.83)	6.20 (5.12)	3.51 (2.87)	4.94 (4.67)	3.57 (3.23)	3.86 (3.99)	1.91 (2.57)
	International, mean (SD)	4.75 (2.99)	3.44 (2.36)	4.25 (2.73)	2.44 (2.43)	7.88 (5.25)	3.28 (2.23)	6.75 (4.44)	2.83 (2.50)	3.00 (2.23)	1.77 (1.87)

Y1, first-year students; Y2, second-year students. *These figures represent the mean size of networks of those students who had indicated a social network. Students who indicated that they had no social network at all are separately described in the text.

#### Social network size comparison between domestic and international students

The comparison of domestic and international students’ social networks showed that first-year international students had larger individual social network sizes than first-year domestic students except for the study-related support network in both the before- and during-pandemic cohorts (see Table 2). Regarding the second year, before COVID-19, international students had larger friendship and information sharing networks than domestic students, but not during COVID-19.

#### Students reporting no social interactions at all

During the pandemic, more students reported that they had no social interactions at all with peers in one or more social network types. Before the pandemic, only two students (2%) reported that they did not have a study-related support network outside of class, six students (6%) reported that they had no collaboration network, only one student (1%) reported that he/she had no friendship network, nine students (9%) reported that they had no information sharing network, and twenty-two students (22%) reported that they had no learn-from network. In comparison, during the pandemic, twelve students (7%), twenty-seven students (16%), twenty-four students (14%), thirty-seven students (22%), and fifty-eight students (35%) reported having no social networks in those five types, respectively.

### Social network structure of first- and second-year medical students

The indegree and outdegree centrality results showed that in “share information” network, the percentages between first- and second-year students were similar for before- and during-pandemic cohorts. It means some students were important sources for sending and receiving information regardless of the influence of the pandemic. However, the other four social networks presented differences between before- and during-pandemic cohorts, and between first- and second-year students. There is less centrality during the pandemic for the social networks such as friendship and collaboration, especially for first-year students (see Table 3). This means that, during the pandemic, first-year students (or influencers) were less dominant in their social networks than the first-year students who started before the pandemic. In contrast, second-year students of the during-pandemic cohort showed similar or even higher centrality than the before-pandemic cohort. This outcome indicates that when students started their studies with social distancing, it is difficult to have influencers in their social networks. Thus, the restrictions seemed to have a stronger negative impact on first-year students than on second-year students.

**TABLE 3:** Indegree and outdegree of first- and second-year students’ social networks before and during the pandemic.

	Centrality	Study-related support		Collaboration		Friendship		Information sharing		Learn-from	
		**First-year**	**Second-year**	**First-year**	**Second-year**	**First-year**	**Second-year**	**First-year**	**Second-year**	**First-year**	**Second-year**
Before-pandemic	Indegree	1.29%	0.42%	1.28%	0.26%	2.14%	0.41%	1.40%	1.43%	2.15%	1.27%
	Outdegree	7.68%	3.47%	4.90%	1.86%	10.68%	2.82%	10.64%	9.93%	14.36%	10.30%
During-pandemic	Indegree	0.31%	1.86%	0.19%	1.29%	0.26%	1.50%	1.38%	0.98%	0.23%	2.24%
	Outdegree	1.39%	6.34%	0.94%	5.09%	1.40%	5.76%	6.14%	7.29%	0.91%	9.39%

### Social network composition of domestic and international students

To investigate how much the international and domestic students mingled, we calculated the average E-I index of students’ social networks during the pandemic (see Table 4). The E-I index indicates whether students were more connected to others with similar characteristics. In general, second-year students tend somewhat more than first-year students toward connecting with peers with similar background characteristics (domestic or international). Meanwhile, domestic students are more likely than international students to connect with peers from similar backgrounds (domestic and international, respectively). It implies that although international students prefer to connect with other international students, international students had relatively more connections with domestic students than vice versa.

**TABLE 4:** Average E-I index of nationality as an attribute to form social networks among first and second year domestic and international students during COVID-19.

	Study-related support		Collaboration		Friendship		Information sharing		Learn-from	
	**D**	**I**	**D**	**I**	**D**	**I**	**D**	**I**	**D**	**I**
Y1 (SD)	−0.745 (0.493)	−0.457 (0.578)	−0.588 (0.595)	−0.425 (0.513)	−0.625 (0.563)	−0.532 (0.563)	−0.621 (0.543)	−0.324 (0.565)	−0.435 (0.604)	−0.285 (0.620)
Y2 (SD)	−0.807 (0.395)	−0.552 (0.411)	−0.746 (0.533)	−0.421 (0.726)	−0.716 (0.446)	−0.505 (0.377)	−0.698 (0.455)	−0.421 (0.529)	−0.479 (0.540)	−0.340 (0.546)

D, domestic students; I, international students; Y1, first-year students; Y2, second-year students.

### Influence of COVID-19 pandemic restrictions on academic performance

Our investigation of medical students’ academic performance before and during the pandemic revealed that - during the pandemic - significantly more first-year students (89.11%) passed the written tests than before the pandemic (83.77%; *p* < 0.05). Although the same tendency was found for second-year students (88.52% vs. 85.19%), this difference was not significant.

The first- and second-year students during the pandemic achieved significantly higher grades [M(SD) 6.54 (1.02), 95% CI (6.44, 6.63) and 6.69 (0.69), 95% CI (6.61, 6.77), respectively) than their counterparts from before the pandemic [M(SD) 6.33 (0.85), 95% CI [6.25, 6.41], and 6.51 (0.65), 95% CI (6.45, 6.58), respectively] (p < 0.001).

### Difficulties students encountered due to the COVID-19 pandemic restrictions and support they need

Based on our thematic analysis to analyze the answers to the open-ended questions: “What kind of difficulties did you meet when you interacted with other students or faculty during COVID time?” and “Do you need any extra support or what kind of extra support do you expect from our medical school?,” we identified three main themes: social connections and interactions, learning and studying, and physical and mental wellbeing. Under each main theme, we identified a corresponding subtheme “support,” and besides, we identified one additional subtheme “communication” under social connections and interactions. In total, 140 students (84% of total participants) reported difficulties, of whom 132 (94%) mentioned social connectiveness and interactions, 37 (26%) mentioned learning and studying, and 14 (10%) mentioned physical and mental wellbeing. We describe the themes below and in Table 5, we support our findings with illustrative quotes.

**TABLE 5:** Quotes illustrating difficulties of students’ interactions during the pandemic and the support they need.

	Quotes
Social connections and interactions	“It was also difficult to truly get to know my fellow students and it stopped me from strengthening these friendships.” (Y1I132) “It is just very hard to maintain friendships with people who I was only just getting close to before the lockdown. It is also quite hard to meet new people since you don’t speak to people during lectures and you cannot just ask people if they want to grab a coffee together or things like that, which is how I would have made friends before.” (Y2D611) “Seeing each other online is very impersonal and I don’t think you “really” know these fellow students and teachers. This is very unfortunate and demotivating.” (Y1D425) Another obstacle is my work in healthcare. I work in a nursing home and because of this I don’t want to take too many risks and I have as few contacts as possible (unfortunately) (Y1D344).
Communication	“It was always nice to meet up with people in between lectures and it really helped to get to meet new friends. Now, it is firstly difficult to meet up and secondly, you have to actively contact others. Automatically, you are not as socially active as you would be if we had not had this situation! “(Y2I575) “There is no room for “small talk” if you don’t see each other outside of lessons.” (Y1D101) “Especially the small talk between lectures has fallen away. We are less up to date with our daily things while that was always very nice to share.” (Y2D771) “All the staff (professors, tutors, etc.) are very welcoming and kind so it felt comfortable and encouraging when interacting with them.” (Y1I86) “The staff does not respond well to e-mails and if you receive one, you have to wait a very long time for a follow-up e-mail or you have to go after it yourself” (Y1D278) “Emailing a professor seemed weird and uncomfortable because I felt like giving them extra-work.” (Y2I659) “It’s hard communicating over video call because of the delay and not being able to see their body language properly.” (Y1I90)
Social support	“Make “social groups” of 3–5 people which change over time, so we could meet people and make new friends.” (Y1I72) “Non-mandatory seminars could be a godsend and a nice stimulus for discussion for students who don’t have many social contacts these days. For example, in the beginning of the pandemic, a “just-chatting” webinar was arranged that was not about a specific topic but where students could just chat with [the teachers].” (Y2D510) “I wished we would have had the option to sign up for something to have daily interaction with a senior student.” (Y2I622) “I hope the faculty organizes something to get to know other students when the COVID rules do not apply anymore. It’s hard to get to know people online or with 1,5m distance. Even when there are some in person events, it’s hard to actually get to know someone since it is usually in a relatively short period of time and the events are all study related, which means you don’t have time to properly talk to someone and get to know them.” (Y1I198)
Learning and studying	“Very annoying that coach groups are not changed. That is currently our only option to meet new people. Instead of rearranging them they are all the same.” (Y1I119) “It is very difficult to have a conversation online, especially during a lecture with 2 fellow students. Then it doesn’t work at all. I mainly made contact after I had spoken to the fellow student at the faculty.” (Y1D165) “It was sometimes difficult to understand each other online, which made the completion of assignments a little difficult.” (Y1D387) “We are expected to do cooperative tasks. This is just very difficult and not fun under these conditions. Online is not ideal.” (Y2D780) “(…) I have to say that I find it very unpleasant to be taught in this way and I am not at all motivated by it.” (Y1D278) “I think you need extra motivation that you often get from other fellow students but now you can’t” (Y2D778)
Educational support	“I really miss the social pressure of the library, and the alternation between social contact and studying. For example, going to a UL [University Library] together, studying for 2h and then getting coffee is much more sociable, than studying alone in your room. I have much more motivation in the CML [Central Medical Library] and UL. I miss support, and by that I mean ways in which the university continues to motivate its students.” (Y2D608) “Yes some extra support would be appreciated such as a more direct contact to certain teachers when looking for help. I feel that e-mail is sometimes ineffective and one to one contact for asking questions is more beneficial.” (Y1D99) “More understanding for internationals, also understanding that they want to be with their family or in their home town especially because they would otherwise often be alone in their small rented room without a lot of contact. Especially if not a lot of friends have been made in the Netherlands more transparency and inclusion when planning to get rid of online exams.” (Y2I659) “Option to decide by ourselves whether to study from home or visit on site education - giving foreign students the possibility to stay in their home countries with their families instead of isolating them” (Y2I610)
Physical and mental wellbeing	“Due to the lack of the social life I felt depressed almost all the time which reflects on my study and assessment results.” (Y1I46) “(…) Often, I feel isolated and alone, even though life with 7 other students (not medical students). (…) In general, COVID has put a strain on my physical and mental wellbeing. I was suffering from health issues before, but the combination of starting in a new city where I don’t know anybody, the stress of studying medicine and COVID made it worse.” (Y1I198) “At first I felt very lost: (1) new country and completely different program (2) my first year as a university student (3) I had no idea what was expected of me and (4) I had absolutely no one to compare myself to at the start. For me this was very frustrating to have absolutely no idea about how I am comparing to the rest of the generation and what I found hard/easy and how studying is going (Y1I86). “I notice that since I moved to Groningen I feel very “alone” there.(…) Because of this, I do notice that I find the uncertain future of my social student life.” (Y1D338)
Mental support	“I would like it if it were easier to get some type of mental support, as I found it really hard to find any specifically for medical students” (Y1D85) “Mental support, we now receive an occasional email which says that they can imagine that studying is difficult at this time, but it is greatly underestimated and such an email does not help me to suddenly feel like doing everything again.” (Y2D780) “Dealing with stress.” (Y1D427) “I need psychological help (and I will get it), and would like to see the UB and CMB always available as workplaces.” (Y2D617)

D, domestic students; I, international students; Y1, first-year students; Y2, second-year students.

#### Social connections and interactions

Due to the pandemic and social distancing, students had no or limited on-site contact with other students, either in or outside of school. Moreover, the students felt they had no other way to meet or contact new people, since everything was closed, like bars, the university, and its libraries. In their opinion, on-site contact is necessary to make new friends. The transition to online learning activities in small groups did not facilitate building social relationships either. In those cases where students did start relationships, the contacts remained superficial or they lost contact with each other. Some did not make any friends at all and others even lost friends. They felt they really had to make an effort to make friends or stay connected because they had to make appointments with peers instead of automatically meeting new friends in college as they used to. Two students also mentioned that because they worked in healthcare, they stopped interacting with others in groups since they did not want to bring risks to their patients. For instance, “Another obstacle is my work in healthcare. I work in a nursing home and because of this I don’t want to take too many risks and I have as few contacts as possible (unfortunately).” (Y1D344).

#### Communication

Communication played an important role in how the students tried to build connections with their peers. They found online communication less personal and missed the small talk between lectures. “There is no room for “small talk” if you don’t see each other outside of lessons.” (Y1D101) When they did contact each other online, most of the time they were talking about academic issues, such as group assignments. Besides, students described that they encountered difficulties in communicating with faculty, which was mostly done by e-mail. Students felt barriers to communicating in this way since they often had to wait a long time for responses from teachers and faculty and they felt uncomfortable mailing to professors and teachers they did not know well. Some students on the other hand felt the contact with teachers and staff was good. They felt welcomed which encouraged them to interact. Besides mail, the students used other communication tools, such as WhatsApp, telephone, and Google meet. Some of them found this helpful and did not need anything else, others felt they had to exert more effort to stay connected.

#### Social support

Although students realized that not much could be done to foster social connectedness due to the COVID-19 restrictions, they offered some nice suggestions to facilitate social support, for instance organizing a free online art course with students from different faculties, with as many on-site sessions as possible as they just wanted activities through which they could meet their peers. Two teachers organized a “chatting” webinar, during which students could just chat with the teachers, which was highly appreciated. As one student mentioned: “Non-mandatory seminars could be a godsend and a nice stimulus for discussion for students who don’t have many social contacts these days. For example, in the beginning of the pandemic, a “just-chatting” webinar was arranged that was not about a specific topic but where students could just chat with [the teachers].” (Y2D510).

#### Learning and studying

Whereas some students thought the online courses and small groups were just fine, others felt that the group learning activities online lacked efficiency and fun. Besides, students thought that the small group composition remaining the same through the semester (where normally this would change two times per semester) hindered them from building new social relationships. Meanwhile, however, some students thought that changing the small group compositions more often may not be good for strengthening their peer relationships. They felt that after they finally had built up bonds with some peers, they, unfortunately, had to switch to another group.

#### Educational support

Students expected the Medical School to offer them more educational support. Some students reported that they actively sought help from the study advisors. Others contacted their tutors. Since students felt they were easily distracted when studying at home, they indicated that opening the University Library would have helped as well to get the motivation to study together with peers and as a place to make friends. Specifically, the international students wished for more understanding and compassion for their situation: far away from home and their relatives. They felt they could not return to their home country, because they had to perform written tests on-site. They would have preferred online exams so that they would have been able to return to their families.

#### Physical and mental wellbeing

Students experienced negative feelings during the pandemic, like loneliness, feeling lost, stress, sadness, and depression. Specifically, first-year and international students reported feeling lost in a new city, with no idea what was being expected from them and without any comparison concerning the performances of their peers. Students thought this affected their studying and study results negatively. For example, a student said: “I notice that since I moved to Groningen I feel very “alone” there. (…) Because of this, I do notice that I find the uncertain future of my social student life.” (Y1D338).

#### Mental support

Students also mentioned they needed psychological or mental support to deal with their emotions. Several reported that they actively sought help by consulting a psychologist.

## Discussion

In this study, we investigated the influence of social distancing on first- and second-year, international and domestic medical undergraduate students’ social network size, structure, composition, and their academic performance. We found that students in the during-pandemic cohort had much smaller individual social network sizes (they contacted fewer peers) than those in the before-pandemic cohort. Social distancing impacted five types of social networks in different ways.

First of all, social distancing restricted students’ social network formation. It reflects the importance of on-site social interactions for peer relationship formation. We noticed that students who had no connections with peers before the pandemic experienced more difficulties in establishing their social networks, particularly friendship networks. When students meet unfamiliar fellow students in the classroom, corridor, or library, they may have a small talk and then start a relationship. But it is difficult for students to have time to start a small talk during online learning, without group activities or faculty’s help, students hardly broaden their social networks, which is common in MOOCs courses (53, 54). Researchers focusing on online learning already tried to facilitate the interaction between students by using online groups or peer feedback activities, such as Facebook groups or audio peer assessment, to improve students’ learning engagement and management, though it still differs from on-site social communication (55, 56). Institutions should notice the differences between on-site and online interactions, especially social interactions, and the importance of providing various online social events for students when they study with social distancing.

The pandemic restrictions limited students’ chances of attending on-site activities and hampered their social network formation, which was reflected in the smaller size of first- and second-year students’ study-related support, friendship, and information-sharing networks during the pandemic than before. The impact of the restrictions on the social network structure was greater for first year students than for second year students. This suggests that second year students could probably more rely on already established networks. Further analysis would require a longitudinal study to follow the further development of networks (57). Although students could contact their peers via social media or online learning activities, they found it hard to get to know others and make friends through online communication. It may be due to students having problems recognizing their social situations when they are not familiar with their fellow students (58). In addition, it is common that students close their cameras and mute their microphones during online education, which leads to the lack of social cues in synchronous online communication and raises difficulties for students to get acquainted with their classmates (59). In a social distancing learning environment, students may prefer to turn off their cameras due to a psychologically unsafe environment (60). Therefore, in this situation, teachers need to spend some time letting students become familiar with the environment and peers, to increase the feeling of safety, and emphasize the role of turning on the camera.

The online learning activities limited the number of peers they met as well, and students found it challenging to maintain relationships this way. Our qualitative findings supported the findings of previous quantitative studies, showing that students experienced mental difficulties, such as feelings of loneliness, depression, stress, and lowered motivation (6, 21–23, 61). Considering that friendship is an important resource of psychosocial support that helps students deal with their negative emotions and stress (15, 62, 63), smaller friendship networks may increase students’ mental health problems. Our qualitative results show that international students may have stronger motivations than the domestics to establish new social networks in a foreign country. However, the social restrictions provided them limited opportunities to do so. The much smaller sizes of international students’ friendship networks during the pandemic compared to before the pandemic may indicate that these students have experienced even more difficulties during the pandemic. For instance, international students experienced unique difficulties: living alone in a foreign country without physical social activities made them long for more understanding and support, which is in line with Elmer’s finding that online communication limits students’ opportunities to maintain and develop new social connections to a great extent (6). Our study reminds institutions to be aware of the diversity of students’ backgrounds and take this into account when designing group activities.

Surprisingly, we found that students’ written test scores in the during-pandemic cohorts were higher than those of the before-pandemic cohorts using the same assessment method. These findings are in contrast with previous findings indicating that students who studied online received lower scores than students who studied on-site (7). Possible explanations for this may be that students had more time to prepare for their exams because they had fewer opportunities to participate in social activities or that students were able to repeatedly watch lectures because they were given online and videotaped. Another explanation may be that medical students facing the change caused by the pandemic came to realize the value of medical knowledge and, therefore, may have been motivated to study. A fourth explanation that is supported by the results of the open-ended questions may be that teachers have delivered the educational content in a more efficient way through online education, which reflects the advantages of MOOCs, or blended learning (64). Mortagy et al. (65) also support the potential benefits of online learning though their national investigation (65). Students’ scores on the written knowledge test indicate that goal direction, one of the three key elements of good learning environments (13, 14, 66), was well taken care of in the online learning setting. Besides, the effectiveness of blended learning was also supported by Vallée et al. (67) through their systematic review. However, both the smaller size and different structure of collaboration networks during the pandemic and the result of our qualitative analysis reflect students’ difficulties in online collaboration in and out of class, especially when they do not yet have peer social networks. To ensure that students thrive in an online learning environment, it is important to enable them to build good relationships and organization/regulation, the other two key elements of good learning environments (13, 14, 66), that play essential roles in students’ wellbeing and development of competencies. Therefore, when students study with social distancing, it is important for institutions/administrations to realize the necessity to build and maintain good relationships between students. They should create some opportunities for students to do social interactions, and revise the formation of group activities in curriculum design to maintain students’ peer interactions.

### Strengths, limitations and future study

The first strength of this study is that we investigated social network formation and academic performance just before the pandemic struck (i.e., under regular conditions where, as usual, group activities took place on site), which allowed us to examine the impact of social distancing measures during the pandemic on students’ social networks formation while keeping the curriculum design otherwise constant. Our study contributes to the understanding of the effect of online learning and social distancing on informal peer relationship formation and students’ learning experience. It will help curriculum designers of online courses notice the importance of improving students’ social interactions to benefit their learning processes. Besides, the fact that the same group activities were conducted in both on-site and online education may contribute to understanding how group activities influence social network formation in both on-site and online education. In addition, we have taken student attributes into account in this study, such as sex, nationality, and grades. Our results may help curriculum designers to develop their program design considering how to improve students’ familiarity through diverse online social activities.

This study has some limitations: First, although academic performance also includes competency performance, we did not include competency development in our study because it is difficult to quantify the assessment of competency development through short-term testing. To have a comprehensive insight into the impact of social distancing on students’ academic performance development and peer relationship formation, future research might also address competency assessment, and further investigate whether there are any long-term effects of the pandemic on students’ academic performance and social network formation. Second, the participants in our study were all from one medical school in the Netherlands. Collaborative research involving multiple medical schools might have led to more robust results. Third, differences in students’ characteristics, such as sex, may also lead to differences in students’ social networks. Future research could further take sex differences into account.

### Practical implications

It is known that communication and social networks should be central elements in a distributed learning environment (68). Understanding the patterns in students’ social network formation during the COVID-19 pandemic may help higher education institutions improve the quality of the online learning environments, in particular by promoting students’ interactions and social network formation (i.e., key element relationships), and ensuring the quality of education.

Drawing from the results of this study, we make the following recommendations:

First, online interactions differ from on-site interactions, particularly on social relationship formation. Therefore, in case of social isolation, we recommend educational institutions to attempt to avoid the lack of social connections by organizing more social events and focusing more on study-related support, friendship, and information sharing social networks when students face social distancing. This also works for online learning courses. If students meet difficulties in peer relationship formation, institutions need to consider more ways inside and outside the formal curriculum to assist students, such as “just-chatting” webinars for students from diverse cultural backgrounds. When these kinds of social activities are still online, organizers need to take measures to increase students’ feeling of safety and make sure all participants turn on their cameras and do not mute during the process.

Secondly, since small online groups were critical resources for students’ social interactions during the pandemic, which also applies to online distance courses such as MOOCs courses, institutions may consider adding various of group activities in curriculum design to maintain students’ peer interactions. Institutions could let students indicate whether they would like to stay in the same group or switch to another group during group activities as well, so tailor the organization more to the students’ preferences.

Thirdly, since students indicated to have experienced problems with their mental health during the pandemic, educational institutions should not only focus on academic performance but also consider wellbeing in a broader sense (24, 69). Educational institutions need to be mindful of their students’ mental wellbeing and provide easy access to find social, educational, and mental support when they need it. Especially for first-year students, formal curriculum and social contacts are important resources for forming peer relationships (12). Besides, institutions should be aware of the diversity of students’ backgrounds, and provide more attention to international students at times when the learning environment is changing dramatically and when distributing students into online learning groups, which is in line with other literature (69, 70).

## Conclusion

This study compared the social network size, structure, and composition of pre-pandemic and during-pandemic cohorts of medical students. The results showed that the size of students’ social networks was smaller during than before the COVID-19 pandemic, especially their friendship network. First-year students perceived more severe challenges than second-year students, and international students experienced a greater impact of the pandemic than domestic students. Although students faced difficulties in social network formation during the pandemic, their academic performance was higher than that of students in the before-pandemic cohort. When institutions are forced to implement blended or online learning during, for instance, pandemics or other situations of force majeure, they need to ensure to create a conducive learning environment by considering social, educational, and mental support to compensate for shortcomings caused by shrinking social networks of their students.

## Data availability statement

The raw data supporting the conclusions of this article will be made available by the authors, without undue reservation.

## Author contributions

YZ collected the data. NB supervised the data collection. YZ and XX organized the data according to the planned analyzes and carried out and wrote the manuscript. AD supervised the especially the qualitative analysis and interpretation. NB, JS-A, AD, and JB improved the data interpretation and revised the manuscript. All the authors made substantial contributions to the research design, the data collection, the interpretation of the data, the manuscript writing, and have also substantively revised it and read and approved the submitted the manuscript.
