# Supplementary material for: Influence of online collaborative learning on social network and academic performance of medical students: lessons learned from the COVID-19 pandemic
Source: Front Med (Lausanne). 2023 Aug 9;10:1242638. doi: 10.3389/fmed.2023.1242638 (PMC10444989; doi:10.3389/fmed.2023.1242638)
Supplement: Supplementary file 1 [file Table_1.DOCX]

Supplementary Material

**Influence of online collaborative learning on social network and academic performance of medical students: lessons learned from the COVID-19 pandemic**

Yan Zhou^1,2†^, Xiaoming Xu^2,3,4†^, Johanna Schönrock-Adema^2,5^, Jasperina Brouwer^6^, Nicolaas A. Bos^2^*, Agnes D. Diemers^2^

† Equal contribution

*** Correspondence:** Nicolaas A. Bos: [n.a.bos@umcg.nl](mailto:n.a.bos@umcg.nl)

# Supplementary Tables

**Table S1** Questions concerning students’ social networks during the pandemic of COVID-19

|  |  | Question |
| --- | --- | --- |
| Social network | study-related support | “In the past semester, when I didn’t understand the study material, I shared my questions or problems that I faced, with this fellow student [name] outside of class.’’ |
|  | Collaboration | “In the past semester, I collaborated with the following fellow students [name] to complete assignments/tasks outside of class.’’ |
|  | Friendship | “whom [name] would you classify as your friend from your fellow students.’’ |
|  | information sharing | “In the past semester, I shared study related information/data with these fellow students [name] outside of class.’’ |
|  | Learn-from | “During the past semester, I have learned a lot from [name].’’ |
| Open questions | How students perceived the impact of COVID 19 on their learning and social networking | “What kind of difficulties did you meet when you interacted with other students or faculty during COVID time?” |
|  |  | “Do you need any extra support or what kind of extra support do you expect from our medical school?” |

**Table S2** Effect sizes of social networks sizes comparison between before- and during-pandemic cohorts

|  | Study-related support | Collaboration | Friendship | Information sharing | Learn-from |
| --- | --- | --- | --- | --- | --- |
| Y1 (*η*²) | .096 | .056 | .150 | .096 | .064 |
| Y2 (*η*²) | .065 | .146 | .129 | .057 | .081 |
